# Disrupted mana and systemic abdication: Māori qualitative experiences accessing healthcare in the 12 years post-injury

**DOI:** 10.1186/s12913-023-09124-0

**Published:** 2023-02-08

**Authors:** John A. Bourke, Helen E. Owen, Sarah Derrett, Emma H. Wyeth

**Affiliations:** 1 Ngāi Tahu Māori Health Research Unit, University of Otago, PO Box 56, Dunedin, 9054 New Zealand; 2 Burwood Academy Trust, Burwood Hospital, 300 Burwood Road, Burwood, Christchurch, 8083 New Zealand

**Keywords:** Qualitative, Indigenous, Māori, Access, Inequities, Quality care

## Abstract

**Background:**

Māori have been found to experience marked health inequities compared to non-Māori, including for injury. Accessing healthcare services post-injury can improve outcomes; however, longer-term experiences of healthcare access for injured Māori are unknown. This paper reports on data from the longitudinal Prospective Outcomes of Injury Study – 10 year follow up (POIS-10) Māori study in Aotearoa/New Zealand (NZ), to qualitatively understand Māori experiences of accessing injury-related healthcare services long-term.

**Methods:**

Follow-up telephone interviews were conducted with 305 POIS-10 Māori participants, who were injured and recruited 12-years earlier, experiencing a range of injury types and severities. Free text responses about trouble accessing injury-related health services were thematically analysed.

**Results:**

Sixty-one participants (20%) reported trouble accessing injury-related health services and provided free text responses. Three related themes describing participants’ experiences were connected by the overarching concept that participants were engaging with a system that was not operating in a way it was intended to work: 1) *Competing responsibilities and commitments* encapsulates practical barriers to accessing services, such as a lack of time and having to prioritise other responsibilities such as work or whānau (family); 2) *Disrupted mana* refers to the feelings of personal disempowerment through, for example, receiving limited support, care or information tailored to participants’ circumstances and is a consequence of patients contending with the practical barriers to accessing services; and 3) *Systemic abdication* highlights systemic barriers including conflicting information regarding diagnoses and treatment plans, and healthcare provider distrust of participants.

**Conclusions:**

Twelve years post-injury, a considerable proportion of Māori reported experiencing barriers to accessing healthcare services. To restore a sense of manaakitanga and improve Māori access to healthcare, Māori-specific supports are required and systemic barriers must be addressed and removed.

## Background

In Aotearoa me Te Waipounamu^1^[1]/New Zealand (NZ), Māori (the Indigenous population of New Zealand) experience greater health comorbidities compared to non-Māori, including higher prevalence of cardiovascular disease [2], chronic obstructive pulmonary disorder (COPD) related hospitalisations [3] and cancer [4, 5]. Māori patients, and their whānau (family), experience greater barriers in accessing primary healthcare [6–14], disease screening [2, 14–16], and secondary care services [16–25] compared with non-Māori. Previous research has also identified practical barriers that hinder Māori patients’ abilities to access primary healthcare, including the cost of appointments and prescribed medications [8–10, 26, 27], availability of transport [13], distance from health services [8, 18], and patients’ time constraints [2, 8, 20]. Structural barriers, such as failure of healthcare centres to offer flexible appointment times, failure to provide continuity of care with a preferred general practitioner (GP), and Māori patients’ past experiences of discriminatory care are associated with delayed disease screening in primary care [7, 10, 12, 17], and often result in more advanced symptoms presenting in public hospitals [2, 8, 17, 27].

Reducing Māori health inequities has been a prioritised goal in healthcare in NZ since the early 2000s. Indeed, under Te Tiriti o Waitangi (a treaty of cession signed between Māori and British Crown representatives in 1840 [28]) the NZ health and disability system is required to achieve equitable health outcomes specifically for Māori. Despite this goal, a Waitangi Tribunal report found that Crown breached Te Tiriti o Waitangi by failing to design and administer a current primary healthcare system which actively addresses persistent Māori health inequities and give effect to the Treaty’s guarantee of tino rangatiratanga (autonomy, self-determination) [29]. Māori continue to experience inequities in access to a range of health services, resources and prescribed medicines [2, 26, 27, 30] given the pervasive, intergenerational impacts of colonisation [31].

Organisational factors that facilitate future healthcare access and disease screening for Māori included delivery of culturally safe, continuity of care from a trusted and often preferred Māori provider [7, 30]. Clear communication from the provider about treatment and health management, and support for the patient and their whānau are also important [4, 12, 14, 17]. In particular, provider-patient whakawhanaungatanga (i.e., a reciprocal relationship of trust, communication and support) is needed to facilitate a culturally safe transition from primary care to hospital services [16]; however, existing structural barriers in a Western-centric health system often make these relationships difficult to establish. Inequities in access to primary healthcare independently account for ethnic disparities in access to specialist curative cancer treatment and cancer survivability, even after adjusting for socio-demographic, cancer type and stage, and comorbidity factors [5, 23, 24].

Māori have also been found to experience marked health inequities after sustaining an injury. Compared to non-Māori, Māori experience twice as much health loss for disability adjusted life years [32], and experience more adverse health outcomes (e.g., pain and psychological distress) at 3- and 12-months post-injury [33, 34]. Our earlier Prospective Outcomes of Injury Study (POIS) study of 2856 New Zealanders injured between 2007 and 2009 (including 566 Māori), found 19% of Māori experienced disability at 24-months compared to 9% pre-injury [35]. Trouble accessing healthcare services after injury was a strong predictor of disability for Māori 24 months post-injury, irrespective of being hospitalised or not as a consequence of the injury [35]. However, trouble accessing healthcare was not associated with disability among hospitalised non-Māori participants [36].

Access to healthcare is an important determinant for positive health outcomes specifically for Māori after injury; however, there is limited research investigating Māori experiences accessing healthcare services specifically for injury. An earlier quantitative survey of primary care users (including 651 Māori), following the implementation of the Primary Health Care Strategy in the early 2000s, revealed that participants experienced less respect, trust, and confidence using Accident Compensation Corporation (ACC) services than other health services [9]. In addition, Māori have lower use of injury-related health services compared to non-Māori [37]. To our knowledge, no studies have qualitatively explored the longer-term experiences for Māori accessing injury-related services, and how access to injury-related services impacts on health outcomes for Māori. This study reports on data from POIS-10 Māori, a longitudinal cohort study of 305 Māori, with a range of injury types and severities in Aotearoa me Te Waipounamu/New Zealand, to qualitatively understand Māori experiences of accessing injury-related healthcare services.

## Methods

The overarching aim of POIS-10 Māori is to understand and identify the factors contributing to long-term experiences and outcomes (positive and negative), for injured Māori and their whānau 12-years post-injury (and 10 years since the last study interviews). Detailed description of the overall POIS-10 Māori design, data collection and analytical approaches have been previously published [39]. However, in brief, this study is underpinned by kaupapa Māori principles, a non-deficit approach which prioritises Māori worldviews and knowledge [40], and inequities, system and structural biases are explicitly investigated [41]. The design of POIS-10 Māori is also explicitly underpinned and guided by key Māori models of health and well-being, e.g. the Meihana Model which requires health providers and researchers to consider the health of a Māori patient (or injured person) in terms of six inter-related components of well-being: tinana (physical), hinengaro (psychological/emotional), wairua (connectedness, spirituality), whānau (family, support people), taiao (physical environment) and iwi katoa (health services/systems of support) [14, 42]. Our study also aligns with Māori data sovereignty principles, in that the data collected is protected and is subject to Māori governance [43]. Māori processes and practices are prioritised throughout POIS-10 Māori; it is Māori-led and the majority of the research team and advisors are Māori (i.e. four of six investigators and six of eight advisors), and two of the four authors of this article are Māori (JB, Ngāi Tahu; and EW, Kāi Tahu, Te Ātiawa, Ngāti Tama, Ngāti Mutunga) importantly, enabling Māori “to have tino rangatiratanga over research that investigates Māori issues” [40] (p. 37).

Māori participants were recruited to the original POIS study via the ACC entitlement claims register, for an injury they sustained between 2007 and 2009. POIS participants who identified as Māori via the NZ census ethnicity question [44] were invited to take part in the new POIS-10 Māori. Interviews were conducted with participants via a structured interviewer-administrated telephone survey (~ 1 hour) using REDCap (Research Electronic Data Capture), a secure web-based management system designed to support data capture for research studies [45, 46]. Highly trained interviewers, the majority of whom were Māori, typed participant responses to the question ‘have you had any trouble accessing any injury-related health services for your injury or any more recent injuries?’ verbatim into REDCap. This question is specific to accessing injury-related health services for the original or more recent injuries, and not in relation to COVID-19.

After completing an interview, participants were posted a thank you card (written in both te reo Māori and English) and a koha (offering; in the form of a voucher) in recognition of participation. Telephone interviews were conducted between March 2020 and June 2021 which was both before, during and after the COVID-19 restrictions. A pause on data collection for 10 days at the start of New Zealand’s nation-wide COVID-19 Alert Level 4 lockdown in March 2020 was taken to allow participants and interviewers to prepare and adjust.

This paper specifically reports on the experiences of injured POIS-10 Māori participants who have had trouble accessing healthcare services. Free text responses were analysed using six iterative thematic analysis phases [47, 48]. First, JB, EW, and SD familiarised themselves with the data (phase 1), before JB grouped data into preliminary codes based on similarities (phase 2). Third, JB actively constructed codes into preliminary themes, which were subsequently reviewed and defined by JB, EW and SD (phase 4 and 5) to ensure themes worked well according to the dataset, codes, and research question. Finally, all authors contributed to interweaving the themes into a narrative, in which participant quotes were used both in an illustrative manner (to highlight key elements of the analysis) and as a basis for analytical comment. Pseudonyms were used for all participants in this manuscript.

## Results

Responses to the access to injury-related health services question were available from all 305 POIS-10 Māori participants, with 61 responding that they reported having trouble accessing injury-related health services and provided free text responses. Ages ranged between 19 and 63 years, with 27 identified as female. Key demographic information about participants who reported having trouble accessing healthcare services are reported in Table 1.

**Table 1:** Characteristics of POIS-10 Māori participants reporting trouble accessing healthcare 12-years post-injury (*n* = 61)

Characteristics	*n* (%)^a^
**Personal**	
Sex	
Male	34 (56)
Female	27 (44)
Age at injury (years)	
18–24	7 (12)
25–34	8 (13)
35–44	21 (34)
45–54	19 (31)
55–65	6 (10)
Adequacy of household income	
Adequate	41 (67)
Inadequate	19 (31)
Living arrangements	
Alone/With non-family	10 (16)
With family	51 (84)
Chronic Health Conditions	
0	7 (12)
1	8 (13)
≥ 2	46 (75)
**Injury-related (collected at recruitment)**	
Injury Severity (NISS^b^)	
1–3 (Least severe)	23 (38)
4–6 (Severe)	26 (43)
> 6 (Most severe)	11 (18)
Perceived threat to life (at time of injury)	
Yes	13 (21)
No	48 (79)
Hospitalised for injury (within week)	
Yes	13 (21)
No	48 (79)

^a^Missing values have not been reported

^b^*New Injury Severity Score*

Three inter-related themes described participants’ experiences and were connected by the overarching concept that participants were engaging with a system that was not operating in a way it was intended to “work”: 1) *Competing responsibilities and commitments* encapsulates practical barriers to accessing services, such as a lack of time and having to prioritise other commitments such as work and whānau (family); 2) *Disrupted mana* refers to the consequences of the practical barriers, namely feelings of personal disempowerment and a subsequent offloading of personal responsibility onto participants to navigate the health system and to fight for what they are legitimately entitled to, such as funding and equipment; and, 3) S*ystemic abdication* highlights systemic barriers that underpin the practical barriers and disrupted mana to manifest in conflicting information regarding diagnoses and treatment plans, and healthcare provider distrust of participants.

### Theme one: competing responsibilities and commitments

This theme encapsulates practical barriers to accessing services, such as a lack of time and having to prioritise other responsibilities such as work and whānau. For some, simply being able to access injury-related health services was not possible due to geographic barriers. Participants who lived in a rural location reported that travel to health appointments could require up to two hours’ travel time, and others experienced trouble having in-home rehabilitation and support equipment delivered. One participant reported how the nature of their work required them to be away from land for long periods of time:

*“I couldn’t really make appointments because back then I was out at sea for 250 days, when back on land last thing I wanted to do was go to the Doctor.”* (David).

For those who might be able to more easily travel to access healthcare services, some described not being able to justify taking time off work and the consequent loss of income. For many participants, maintaining their income and whānau responsibilities were, in reality, far greater priorities than attending injury-related healthcare and follow-up appointments:

*“I should have tried to get more help,* [but at the time] *I was trying to work and look after my kids* [so accessing health services] *took the back burner.”* (Hamuera).

In addition to barriers within participants’ personal environments, there are numerous instances of participants having difficulty securing a timely healthcare appointment. Trying to align what limited availability participants had in their personal life with the often sporadic and unpredictable health service was problematic:

*“Trying to get a* [healthcare] *appointment when you need it is difficult …*[and I often] *have to go for days without an appt…sometimes* [I] *tried 20 different physios but couldn’t get in immediately…I applied for home help twice and it was going to take 3 weeks to get someone organised.”* (Kelly).

Such an example of delayed access to services negatively impacted on participants’ injury recovery experiences and perceived feelings of support from healthcare providers. Taken together, the variety of barriers participants reported experiencing when accessing healthcare services for injury, whether location, financial, or time-specific constraints due to work or childcare, paint a picture of people who have a large number of competing commitments in their life. The added complexity of inconsistent availability of healthcare providers further challenged participants, who found themselves in an often exhausting and constant cycle of negotiating a variety of equally important but compromised life responsibilities.

### Theme two: disrupted mana

The second theme, *disrupted mana*, refers to the personal toll of negotiating numerous, and often repeated, barriers to accessing existing healthcare services. Barriers could manifest as ableism such as a lack of wheelchair access, or not being able to access a driver or suitable transport to travel to healthcare providers, speaking to the idea that certain accessibility requirements and support services are critical to ensuring people can access healthcare services:

“[I often have] *trouble accessing places as they don’t have wheelchair access,* [I] *could go elsewhere, but [I] need a driver and it is a hassle.”* (Rachel).

However, barriers could also be experienced as an inconsistency regarding who they saw, which resulted in feelings of frustration, as one participant recalled:

“[I have such] *trouble accessing the same person!!* [I] *can never see the same GP because they keep changing so I have to tell the whole story again and I hate that.”* (Steve).

Such experiences served to compromise the integrity of a person’s mana.^2^ For example, a lack of support and a scarcity of whānau-centred care resulted in situations where negative interactions with health services were blamed on the injured participants:

*“At the hospital, it was meant to be a day surgery but kept me in overnight and found my stay was unnecessary. On [statutory holiday] the doctors went on holiday and the nurses were upset with me being there but there were no doctors! They wouldn’t give me crutches and I had to walk to the bathroom so I ended up wetting the bed which was very humiliating and frustrating…when I finally had a doctor approve my discharge they basically kicked me out, even though they were the ones that had insisted I stay!”* (Kaia).

Such experiences, combined with instances of being transferred from provider to provider, or not receiving tailored support or funding were exhausting for participants, and instilled a sense of disempowerment and subsequent and seemingly expected (by healthcare services) personal responsibility for managing their own healthcare. An unnecessary consequence of personal responsibility was having to self-advocate and follow-up on treatments, support and funding. For example, one consequence of feeling disempowered and isolated was participants feeling the need to initiate things themselves. Several participants often funded private treatment and equipment. Of course, that option was only available to those who had the means to do so. As one participant said,

*“I bought my own crutches - so I could have my own as I used them for a long time. My family bought a hard mattress that really helps my back.”* (Tayla).

While it appeared that participants were capable of such self-reliance, it caused additional stress and was exhausting, and fundamentally reflected that the health system was not operating or providing care as it was designed to.

### Theme three: systemic abdication

The third theme examines some of the more systemic and structural barriers that participants reported when trying to access healthcare services for their injuries. For example, participants often reported hearing confusing and conflicting information regarding their diagnosis and treatment plans, and delayed access to overloaded health services. For example, one participant described repeated trips to the GP following a back injury. Over a space of nearly two years, the participant experienced several misdiagnoses, several very complicated surgeries, and was referred to a number of different medical specialists. Further concerns about the navigation and clarity of paths to follow-up healthcare and support after their injury were reported by participants. For example, one participant said,

*“I got ACC [financial support] but no further information…this was not ok as I had severe concussion and could have done with some help [I didn’t know] what to do next.”* (Peter).

Other participants recalled similar experiences, of “being discharged with no follow-up*”,* and the compounded negative impact of receiving inconsistent injury-related compensation, and the flow-on effects:

*“Getting ACC was a nightmare for the first injury in 2009, back then I remember getting about $100 a week and my rent was $85 a week I had to try and survive on $15 a week, it was awful. The second recent injury, my payments would stop every now and again. This was stressful as I needed the payments regularly, if I didn’t ring then I believe they wouldn’t have restarted. Apart from two weeks of home help in my initial injury I have had to pay for everything else myself.”* (Rawiri).

Such an example demonstrated the “real” impact of inadequate access to, and provision of, healthcare services and the subsequent need for participants to be self-reliant and take responsibility for their own healthcare. This was arguably not how the healthcare system in New Zealand was meant to work. Perhaps of most concern, some participants were made to feel unworthy of the treatment they received: services that as New Zealand citizens, they were legitimately entitled to:

*“Within the health system I was really challenged on why I was given the surgery…the doctor said that lots of sportsmen could take my place that have a professional career ahead of them…I had to stick up for myself, and say that my life was important too. He said I was taking someone else’s place. I wasn’t worthy of it. I had to work hard on rehabilitating it before the operation so that I was in the best state before surgery to have the best outcome.”* (Matiu).

Such examples of when systematic barriers become personal were reported by several participants, for example, instances of discriminatory interaction when assumptions were made regarding the aetiology of one’s injury:

*“I’ve got a scar on my shoulder and the surgeon told them that I had been in a knife fight. He was an idiot, I thought maybe it was because I was Māori he thought it might have been the case.”* (Henare).

Such experiences speak to discriminatory profiling, of negative stereotyping or ‘assuming’, representing not only an incredibly devaluing personal experience, but an example of how systematic and implicit cultural alienation manifests as overt discrimination at the level of human interaction. In addition, such interactions do not only devalue a person, but reflect the clear absence of support and recognition from a system that was designed to, but was not there to help or support all those injured.

## Discussion

Despite efforts to reduce Māori health inequities, the Aotearoa me Te Waipounamu/New Zealand health system is still structured upon dominant European cultural beliefs and values. For example, the lack of consideration of rongoā (traditional medicinal applications and treatments) and lack of information for, or acknowledgement of, the important role of whānau in facilitating positive health outcomes [30], illustrate a mismatch between service delivery and Māori practices and needs [17]. Findings in this study suggest that during the 12 years post-injury, POIS-10 Māori participants reported experiencing various barriers to accessing healthcare, ranging from practical barriers such as geographic location to nearest health centre, travel time required, and time constraints of work and childcare, which often took precedent over injury-related needs. Indeed, Māori women with children are less likely to attend a GP or secondary care appointment if they are offered unsuitable times (e.g., after-hours care at a greater cost), if whānau are unavailable for child-sitting, or if their children’s health needs are greater [8, 17].

Of considerable concern, are the systemic barriers participants faced such as not receiving tailored support, care or information, and conflicting information regarding diagnosis and treatment plans. This lack of clarity around treatment pathways can result in patients’ reluctance to ask questions, leaving them feeling disempowered, disillusioned and misinformed about the best course of action for their healthcare, and consequently, being less likely to attend future appointments [16]. Furthermore, participants experienced provider distrust regarding the cause of their injuries, and their worthiness and right to receive certain healthcare services were questioned. Together, such long-term barriers to accessing health services gave rise to participants feeling personally disempowered and resigned to having to personally advocate and pay for healthcare services that should be provided by the publicly funded health system and no-fault injury insurance scheme (i.e. ACC). These barriers cumulatively contribute to the overarching concept that participants were engaging with a system that was not operating in a way it was intended to “work”.

Participants in this study repeatedly reported feeling under-supported by health services and facing barriers such as inconsistent and irregular clinical appointments, even though participants varied in terms of physical health, comorbidities and hospitalisation status. Previous research has also highlighted how Māori patients often face structural barriers to accessing primary health care, with existing models of care consistently not meeting their needs [2, 10, 17, 31]. It has been reported that, compared to non-Māori, proportionately fewer Māori were given their preferred appointment times, were offered a choice of times, or were able to see their choice of GP [10, 12]. For those who were given a suitable appointment time, Māori patients were less likely to be seen on time [10] and for shorter durations [5]. These access inequities are partly attributed to Māori ‘self-silencing’ for fear of ‘being a nuisance’, and having lower expectations for what constitutes satisfactory care [10, 11]. The need for flexible appointment times is particularly crucial for Māori women who report balancing childcare and work responsibilities and have limited spare time [2, 8, 20]. Concerningly, these barriers to Māori mothers also prevent the receipt of secondary treatment for known chronic illnesses (e.g., rheumatic fever) [12].

Furthermore, and concerningly, the findings of the study show that culturally unsafe interactions between healthcare providers and Māori patients are common and include negative ethnic profiling, being made to feel personally responsible for any negative interactions and unworthy of the treatment they require to the extent that they felt like a burden to the public health system. This supports previous research findings that even when Māori *can* access health services, they are more likely to receive discriminatory care and experience lower quality interactions with providers than NZ Europeans [6, 31]. Evidence from qualitative studies reveals that Māori are more likely to experience provider-centric, culturally unsafe care during fragmented appointments with unfamiliar providers [3, 6, 7, 12, 17]. Experiences of culturally unsafe care include a failure to acknowledge patients’ psychosocial needs [3, 17], to provide information about prognosis and treatment [4, 7, 9], and to establish rapport with patients and their whānau [6, 7, 11, 12]. Also, due to knowledge limitations, providers commonly disregard traditional Māori practices and healthcare, even as a supplementary or palliative care approach (e.g., mirimiri massage and rongoā) [7, 30]. Culturally unsafe care increases patient fear and uncertainty about their health and treatment pathways in both primary [6, 7, 30] and secondary care settings [15, 16]. Māori patients who have had previous negative experiences with their provider are less likely to attend cancer or rheumatic fever screening tests or schedule an appointment if they have symptoms [4, 12].

### Implications and recommendations for health providers

To facilitate improved Māori access to healthcare services and ameliorate many of the barriers identified above, service delivery should align with Indigenous frameworks, such as the Meihana Model, to ensure culturally safe and high quality care is designed and received, including for longer-term injury rehabilitation [42, 49, 50]. The Meihana Model is a clinically relevant framework which supports health professionals to work effectively with Māori and their whānau [50]. It asserts that healthcare should be delivered through a four-stage hui process [51]: mihimihi (initial greeting to start engagement or appointment), whakawhanaungatanga (to build the relationship), kaupapa (purpose of appointment and patient/whānau needs), and mihi whakamutunga (explanation, planning and closing of appointment).

At the inter-personal level, if health providers use Indigenous frameworks this will help ensure provider-patient interactions are respectful, culturally safe and focus on relationship building [3, 51]. Health providers should enable whānau involvement in consultations to gain a more comprehensive understanding of the patient’s medical history, how whānau can help manage and navigate the health system with the patient, and what support networks are available. The involvement of whānau is particularly important given that 84% of our sample lived with whānau. In previous research, Māori reported that their regular GP caring about well-being and wider whānau helped overcome fears of the health system and increased their willingness to engage with the treatment plan [7, 30]. Additionally, healthcare agencies could consider including KPIs into the work requirements of non-Māori healthcare workers, and appointing more Māori to high-level positions in healthcare agencies to oversee services to Māori.

At the organisational level, more general practices and specialist facilities should allocate more time and resources to professional development opportunities to increase culturally safe practice of non-Māori health professionals. Health providers should use Māori frameworks to plan initiatives or make evidence-based decisions for their organisation to achieve better outcomes for Māori and reduce inequities [40, 42]. Additionally, practical barriers to care for patients, for instance, transport, car park spaces, and additional seating for whānau should be rectified [42]. To support culturally safe healthcare, organisations could “match” providers to patients to facilitate connections to services and provide further support to whānau [11, 13]. Supporting this, Heke et al. [52] argue for the development of universal cultural competence standards to deliver culturally responsive health services. Patients are more likely to experience culturally safe care if they receive continuous healthcare and relational support from one trusted (ideally) Māori provider [7, 26]. Continuity of care is associated with effective communication [11], clear information provision (e.g., health management, disease profiling, treatment) [4, 12, 17, 26], trust, and equal access to disease screening [2, 12].

At a systems' level, our findings highlight the importance of eliminating inequitable treatment practices and the need to develop evidence-based, equity-focussed healthcare systems that will improve understanding and response to health needs and outcomes for Māori. Achieving such change requires Kaupapa Māori, co-design approaches [53] to ensure Māori receive high quality and culturally safe care and experience provider-patient whakawhanaungatanga [16, 23]. Such initiatives would align with Te Aka Whai Ora, a new statutory entity to ensure the New Zealand health system is grounded in te ao Māori and ensure that the wider health system better recognises and is more responsive to Māori needs [54]. Scott [49] states that sometimes people want to locate the explanation for inequities with the individual, when often it is the systems that have the most significant impact and must provide high quality equitable healthcare to reduce inequities. Our findings highlight practical, organisational, and systemic barriers to healthcare access rather than unresponsive individuals. Therefore, systems must be responsive and flexible to the needs of Māori.

## Conclusion

To our knowledge, our study is the first to investigate Māori experiences of accessing healthcare post-injury over a 12-year period. Māori not only experience practical barriers to accessing healthcare but also systemic barriers through culturally unsafe interactions with their provider, their healthcare centre, and when navigating the health system. Further research is required to explore Māori perceptions of factors that helped them access healthcare services, several years following an injury event, and to further understand the impacts of trouble (or ease) of accessing healthcare services on the health and well-being of Māori long-term. To restore a sense of manaakitanga (generosity and care) and improve Māori access to healthcare care, Māori-specific supports are required.

## Data Availability

The datasets generated and/or analysed during the current study are available from the corresponding author on reasonable request.

## Abbreviations

ACC: Accident Compensation Corporation (ACC)
NZ: Aotearoa/New Zealand
COPD: Chronic obstructive pulmonary disease
NISS: New Injury Severity Score
POIS: Prospective Outcomes of Injury Study
POIS-10: The longitudinal Prospective Outcomes of Injury Study – 10 year follow up
GP: General practitioner

## Footnotes

1 All translations and meanings in te reo Māori have been informed by Te Aka Māori Dictionary (see references for details)

2 (noun) prestige, authority, control, power, influence, status, spiritual power, charisma - mana is a supernatural force in a person, place or object. https://maoridictionary.co.nz/search?idiom=&phrase=&proverb=&loan=&histLoanWords=&keywords=mana
